# A rare anatomic variation of the superficial palmar branch of the radial artery causing pain

**DOI:** 10.1007/s00276-017-1936-6

**Published:** 2017-11-09

**Authors:** Georg Singer, Robert Marterer, Holger Till, Barbara Schmidt

**Affiliations:** 1 Department of Pediatric and Adolescent Surgery, Medical University of Graz, Auenbruggerplatz 34, 8036 Graz, Austria; 2 Division of Pediatric Radiology, Medical University of Graz, Graz, Austria

**Keywords:** Radial artery, Adolescent, Pain, Abductor pollicis brevis

## Abstract

**Background:**

The superficial palmar branch of the radial artery (SPBRA) normally pierces through the thenar muscles and unites with the ulnar artery to form the superficial palmar arch. Rarely, a subcutaneous course of the SPBRA is described in which the artery lies superficial to the thenar muscles.

**Case report:**

We report about a 17-year-old female patient with pain at the thenar eminence due to a unique course of the SPBRA. Duplex sonography and magnetic resonance angiography revealed a subcutaneous course of the artery over the thenar muscles. Arterial transposition by splitting of the abductor pollicis brevis muscle was performed. At 12-month follow-up, the patient is still free of symptoms. Duplex sonography confirmed patency of the SPBRA.

**Conclusion:**

While a subcutaneous course of the SPBRA has been described before, we present an adolescent patient with this anatomical variation causing pain. Our specifically tailored treatment strategy consisting of arterial transposition by splitting of the abductor pollicis brevis muscle was efficient and feasible in our patient and hand surgeons should be aware of this anatomical variation.

## Introduction

The superficial palmar branch of the radial artery (SPBRA) arises from the radial artery at the level of the distal forearm. After passing the carpus, the vessel normally pierces through the thenar muscles to unite with a branch of the ulnar artery to form the superficial palmar arch [6]. Nevertheless, a number of different variations in size, branching patterns and course of the SPBRA have been reported [4]. Rarely, a subcutaneous course of the SPBRA is described in which the artery lies superficial to the thenar muscles [4]. Usually this rare variation does not causes clinical complications, but the present case report describes a 17-year-old female patient with repetitive pain located at the thenar eminence. The radiological anatomy as well as the surgical procedure and its outcome are presented.

## Case report

**Fig. 1:**
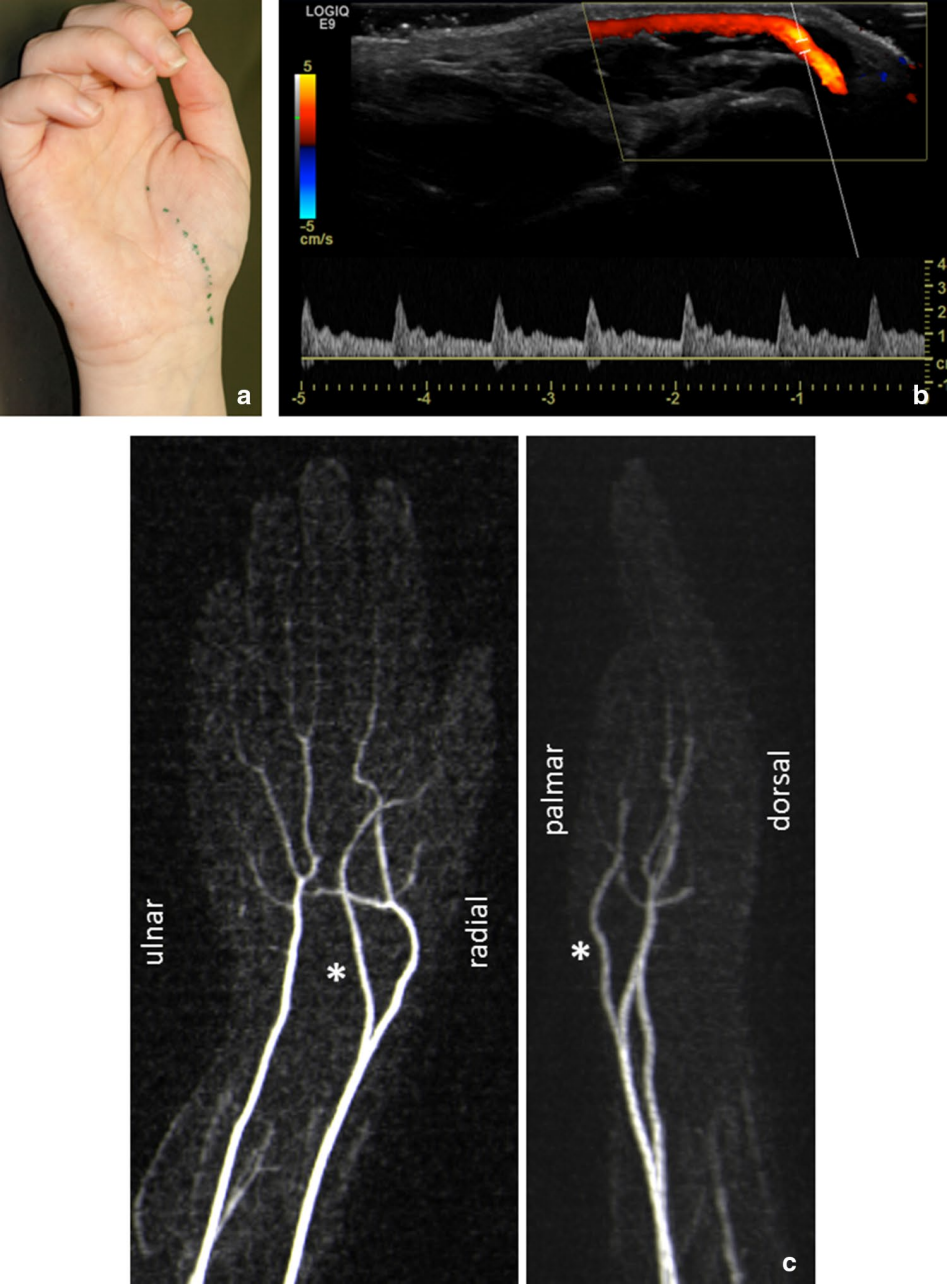
Palpable course of the superficial branch of the radial artery (**a**). Duplex sonography revealed a subcutaneously located vessel with arterial flow pattern (**b**). Magnetic resonance angiography demonstrated a large calibered SPBRA with a superficial course (asterisk, **c**)

A 17-year-old female patient was admitted to our outpatient clinic with pain located over the right thenar eminence since 6 months. The pain became worse when grasping objects. No history of trauma could be elicited. Clinical examination revealed free range of motion of the wrist joint and unremarkable peripheral blood flow and motor function without paresthesia of the fingers. Nevertheless, a pulsating vessel could be felt over the thenar eminence (Fig. 1). The patient described pain when pressure was applied directly onto the vessel. Sonography showed a subcutaneously located vessel with arterial perfusion pattern (Fig. 1). A magnetic resonance angiography was performed revealing a large calibered SPBRA with a superficial course and the absence of the superficial palmar arch (Fig. 1). Splinting for 2 weeks did not cause symptom relief. Since the pain persisted for another 2 months, an operative procedure consisting of arterial transposition was suggested.

**Fig. 2:**
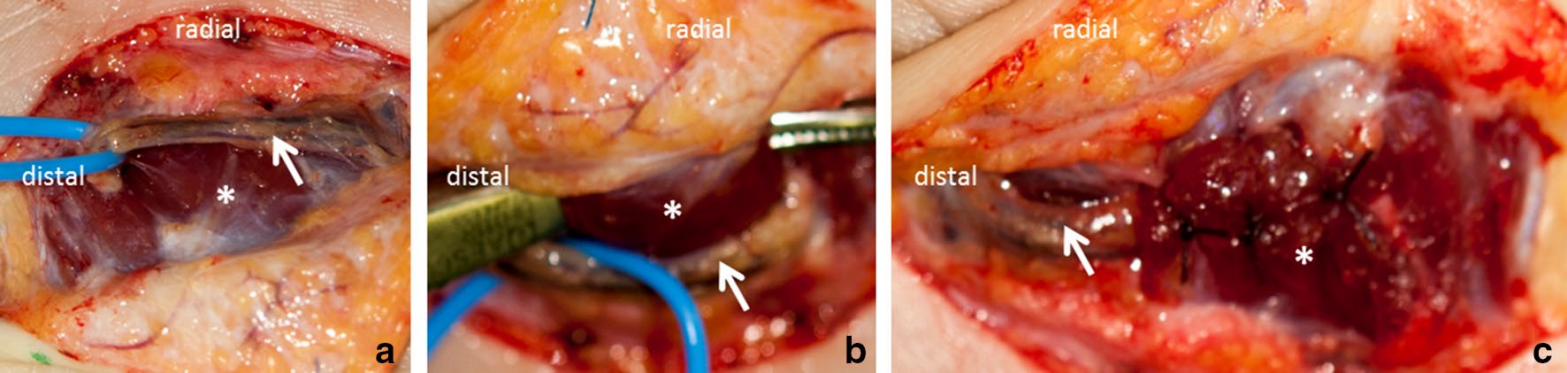
Intraoperatively, the SPBRA with accompanying veins (white arrow) was located subcutaneously superficial to the abductor pollicis brevis muscle (asterisk) (**a**). The muscle was tunneled using a curved clamp (**b**). Following muscle dissection the SPBRA was transpositioned to lie within the thenar muscles (**c**)

Due to the absence of information about a superficial course of the SPBRA causing pain in the literature, we have specifically tailored the following approach: an incision was made along the thenar crease. Following elevation of the skin flap, the macroscopically unremarkably appearing SPBRA could be located within the subcutaneous tissue over the abductor pollicis brevis muscle (Fig. 2a). The muscle was tunneled using a curved clamp (Fig. 2b) and carefully dissected using cautery. Thereafter, the artery was transposed beneath the muscle. The muscle was re-approximated using interrupted absorbable sutures without compressing the SPBRA (Fig. 2c).

Postoperatively, a splint with adduction of the thumb was applied. The postoperative course was uneventful. The skin sutures and the splint were removed after 2 and 4 weeks, respectively. At latest follow-up, 1 year following the surgical procedure, the patient was free of symptoms with free range of motion of the thumb joint and no signs of weakness of the M. abductor pollicis brevis. Sonography showed a patent vessel beneath the abductor pollicis brevis muscle.

## Discussion

The present report describes a case of an adolescent patient with a superficial course of the SPBRA causing pain. The artery was located superficial to the thenar muscles. Our specifically tailored approach consisting of arterial transposition by splitting of the abductor pollicis brevis muscle led to complete resolution of symptoms.

The arterial blood supply of the hand has been the focus of a myriad of anatomical and radiological studies [1, 2]. Anatomic variations of the major arteries of the upper extremity have been reported in about 10% of individuals [7]. Normally, the SPBRA pierces the thenar muscles, which it supplies, and anastomoses with the end of the ulnar artery to form the superficial palmar arch [6]. A number of variations of the branching patterns, size and course of the SPBRA have been reported in the literature. Tagil and colleagues, for instance, have described a SPBRA traveling transversely over the flexor retinaculum and joining the ulnar artery without reaching the thenar muscles [6]. Another case report presents a complex variation in the pattern of blood supply to the palm of the hand including the SPBRA coursing superficial to the thenar muscles similar to our patient [4]. However, this variation was found during a routine dissection including both upper extremities of a 52-year-old male cadaver. While in both cases the SPBRA had a similar superficial course, the superficial palmar arch was absent in our patient.

The vast majority of the reports describing variant courses of the SPBRA is confined to anatomical post-mortem studies. Therefore, it can be assumed that irregular courses of the artery seldom cause clinical problems necessitating intervention. Kokkalis and coworkers, however, have described a case of a patient with an aberrant SPBRA [3]. An 80-year-old man presented with a 5-year history of right hand carpal tunnel syndrome. Open surgery revealed an artery running from radially to medially, almost parallel to the median nerve. The artery originated from the radial artery approximately 1 cm above the wrist crease and ended at the superficial palmar arch [3].

To the best of our knowledge, a superficial course of the SPBRA causing clinical problems and therefore no treatment strategy has been published yet. External compression of the superficially located SPBRA and subsequent partial ischemia could be explanation of the pain which aggravated when grasping objects in our patient. These dynamics of arteries of the hand are supported by a report of Ritz et al. who have examined 91 radial and ulnar arteries in 23 volunteers by color duplex ultrasonography. The authors found that 11% of superficial palmar branches of radial arteries occluded when clenching a fist [5]. However, all included subjects were asymptomatic at the time of examination. Therefore, the exact underlying pathophysiologic reason of the pain in our patient remains unclear.

Since it is described that ligation of the SPBRA should be avoided because of its blood supply to the thenar muscles and to the palmar arch, except in the case of large aneurysms [8], transposition of the artery by splitting of the abductor pollicis muscle without compromising the SPBRA seemed to be the best treatment option for our patient. To facilitate healing of the muscle, we have decided to apply a splint with adducted thumb for 4 weeks.

In conclusion, we present a case of an adolescent patient with an anatomical variation of the SPBRA with a subcutaneous course over the thenar muscles causing pain. Arterial transposition by splitting of the abductor pollicis brevis muscle was efficient and feasible in our patient and orthopedic and hand surgeons should be aware of this possibility.
